# Osteomyelitis of the Mandible Caused by Late Fracture following Third Molar Extraction

**DOI:** 10.1155/2019/5421706

**Published:** 2019-08-04

**Authors:** Shinsuke Yamamoto, Naoki Taniike, Daisuke Yamashita, Toshihiko Takenobu

**Affiliations:** ^1^Department of Oral and Maxillofacial Surgery, Kobe City Medical Center General Hospital, Kobe, Hyogo 650-0047, Japan; ^2^Department of Pathology, Kobe City Medical Center General Hospital, Kobe, Hyogo 650-0047, Japan

## Abstract

The common postoperative complications of the extraction of third molars are frequently reported; however, reports about osteomyelitis of the mandible caused by late fracture following third molar extraction are rare. Here, we report a case of osteomyelitis of the mandible caused by late fracture following third molar extraction. A 38-year-old Japanese man was referred to the surgery department with chief complaints of dull pain and swelling in the right masseteric region and paresthesia of his lower lip and mental region in March 2018. A family dentist removed his lower third molar in the right side in January 2018. When the patient was chewing an innards stew 23 days after the procedure, he heard a cracking sound from the right mandible. Thus, we diagnosed the patient as having osteomyelitis of the mandible caused by late fracture following third molar extraction and performed sequestrectomy and curettage under general anesthesia in April 2018. In conclusion, it is necessary to recognize the possibility that late fracture following third molar extraction can cause osteomyelitis. Furthermore, once osteomyelitis of the mandible caused by late fracture occurred, early and appropriate treatment is necessary because the osteomyelitis may progress rapidly and in some cases may result in pathological fracture.

## 1. Introduction

The common postoperative complications of third molar extraction are alveolar osteitis, secondary infection, bleeding, and paresthesia [1–10]. On the other hand, major complications, such as mandibular fracture, severe hemorrhage, or iatrogenic displacement of the third molar teeth, are rarely reported [6]. Among them, the incidence of mandibular fracture during or after a third molar extraction has been reported to be 0.0046% to 0.0049% [11, 12].

We report herein a case of osteomyelitis of the mandibular caused by late fracture in a Japanese man.

## 2. Case Report

**Figure 1:**
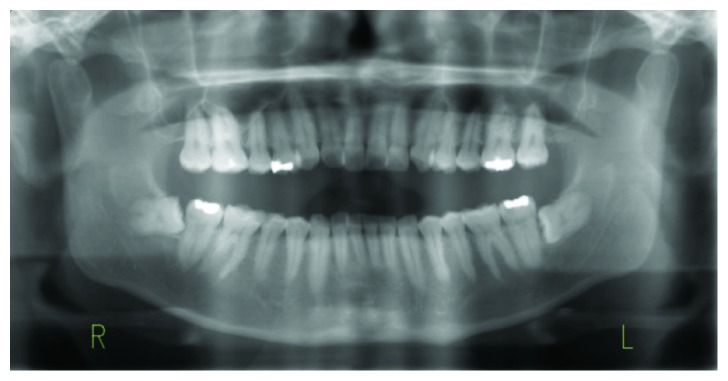
A panoramic radiograph obtained before the extraction. The radiograph shows that the right lower third molar was horizontal (Pell and Gregory Class I, position A). There are no prior bone lesions associated with the molar, such as cyst and tumor.

**Figure 2:**
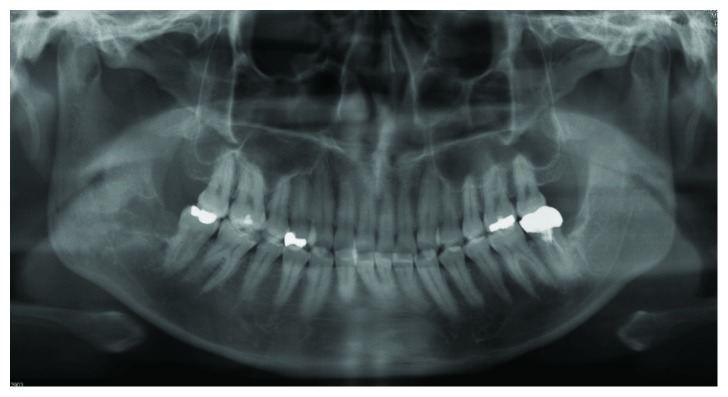
A panoramic radiograph obtained during the initial visit. The radiograph shows radiolucency from the extraction socket to the right mandibular angle.

**Figure 3:**
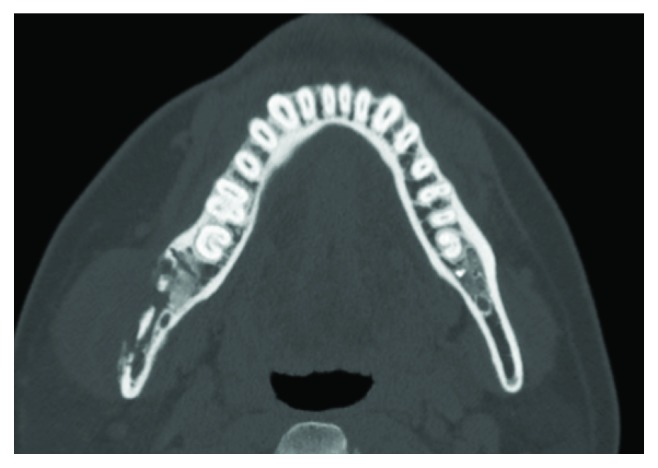
Coronal computed tomography (CT) scans obtained during the initial visit. CT shows a destruction of buccal cortical bone from the socket to the right mandibular angle and a formation of bone sequestrum. Although the lingual cortical bone is intact, there is periosteal reaction around the buccal bone destruction.

**Figure 4:**
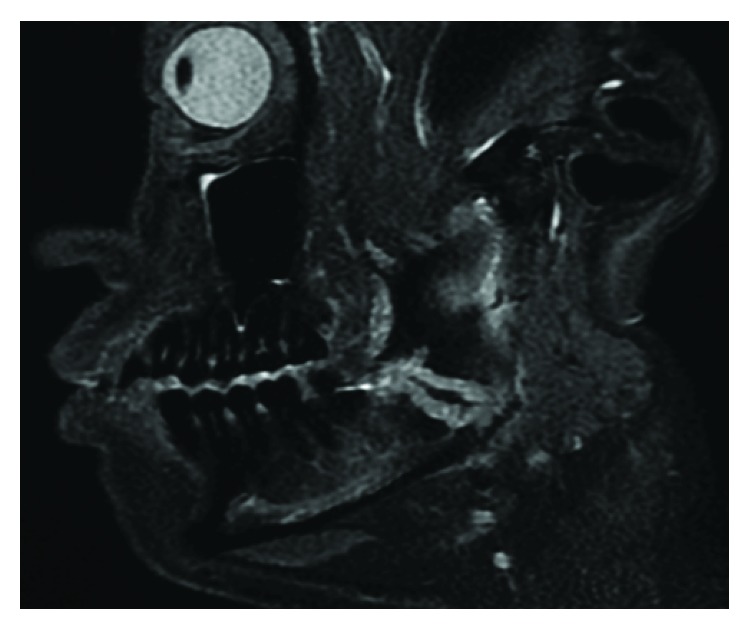
Sagittal magnetic resonance imaging (MRI) obtained during the initial visit. MRI shows that the mandibular bone marrow from the right ramus to the body has a high signal density on short-tau inversion recovery (STIR) images and the cortical bone is partially raptured. Moreover, filaments in the low-density area are observed in the high-density area.

**Figure 5:**
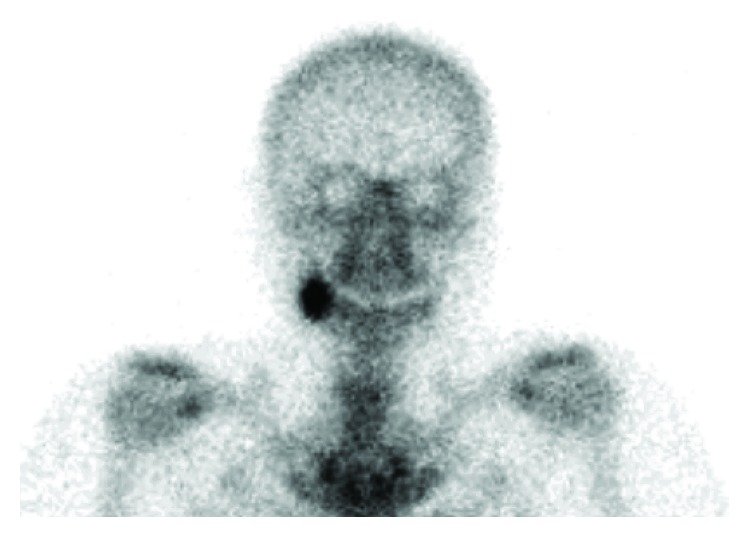
Bone scintigraphic findings obtained during the initial visit. Bone scintigraphy shows a strong accumulation of technetium medronic acid (99mTc-MDP) in the right mandibular ramus.

A 38-year-old Japanese man was referred to the Department of Oral and Maxillofacial Surgery at Kobe City Medical Center General Hospital with chief complaints of dull pain and swelling in the right masseteric region and paresthesia of his lower lip and mental region in March 2018. He had no medical history and was not taking any medication. His lower third molar in the right side was removed due to pericoronitis under local anesthesia by a family dentist in January 2018. The tooth was partially and horizontally impacted (Pell and Gregory Class I, position A) (Figure 1). The method of the extraction involved local anesthesia administration, surgical incision, elevation of a mucoperiosteal flap, ostectomy, tooth sectioning, dislocation, curettage of granulation tissue, irrigation with saline, and suturing. The surgical procedure was uneventful, and he was administered amoxicillin for 3 days. At 7 days after the procedure, he only had mild postoperative pain and had no paresthesia of the lower lip and mental region, as confirmed by his family dentist. At 23 days after the procedure, when the patient was chewing an innards stew, he heard a cracking sound from his right mandible and felt mild pain, and his right masseteric region began to swell. The dentist found no abnormalities on his occlusion and panoramic radiograph. However, he was placed on a soft diet; to make matters worse, he had paresthesia of his lower lip and mental region in the right side in addition to worsening of the pain and swelling at 42 days after the procedure. Treatment with mecobalamin and adenosine triphosphate disodium hydrate was initiated, and he was referred to our department for further examination of paresthesia and pain at 55 days after the procedure. Clinical examination at the initial visit showed mild swelling at the right masseteric region, mild paresthesia of his lower lip and mental region in the right side, and trismus (25 mm). Mandibular movements were normal, and there were no observable changes in the dental occlusion. Electric pulp test revealed that mandibular first and second molars of the right side were vital. The alveolar mucosa of mandibular third molar extraction socket was healed, but there was mild tenderness, and the distal periodontal pocket of the second molar was deep and hemorrhagic. Blood tests showed a white blood cell count of 4,900/mL (range, 3,900 to 9,800/mL) and C-reactive protein level of 0.38 mg/dL (range, 0.00 to 0.50 mg/dL). Panoramic radiograph showed a radiolucency from the extraction socket to the right mandibular angle (Figure 2). Computed tomography (CT) showed destruction of the buccal cortical bone from the socket to the right mandibular angle and formation of bone sequestrum. Although the lingual cortical bone was intact, there was periosteal reaction around the buccal bone destruction (Figure 3). Magnetic resonance imaging (MRI) revealed that the mandibular bone marrow from the right ramus to the body had a low signal density on T1-weighted images and a high signal density on short-tau inversion recovery (STIR) images (Figure 4). Bone scintigraphy showed a strong accumulation of technetium medronic acid (99mTc-MDP) in the right mandibular ramus (Figure 5).

**Figure 6:**
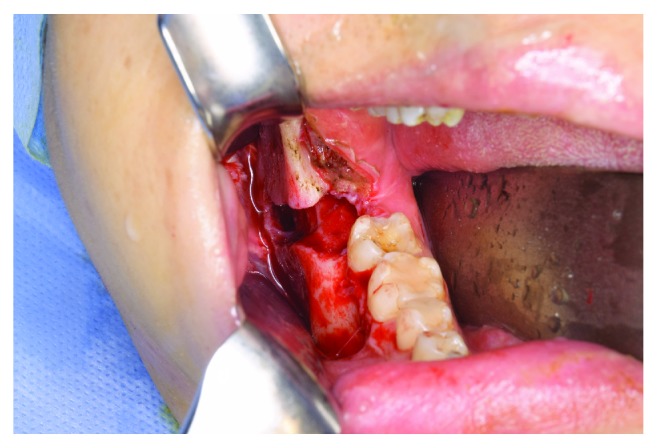
An intraoperative photograph. The size of the defect of the buccal cortical bone was 25 mm (height) × 8 mm (width) × 6 mm (depth), which reached from the extraction socket to the right mandibular angle.

**Figure 7:**
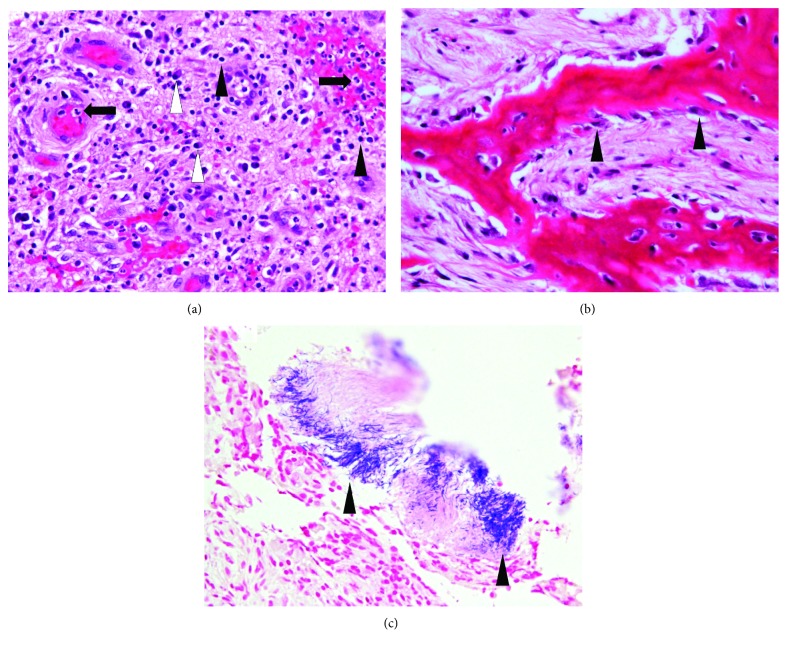
Surgical specimen of the granulation tissue. (a) The majority of the surgical specimen is granulation tissue composed of lymphocytes (black arrowheads) and plasma cells (white arrowheads), with neutrophil infiltration (black arrows) (H-E, ×400). (b) The surgical specimen includes neonatal bone accompanied by many osteoblasts (black arrowheads) (H-E, ×400). (c) Colonies of *Actinomyces* sp. (black arrowheads) are recognized in the inflammatory infiltrates (Gram, ×400).

**Figure 8:**
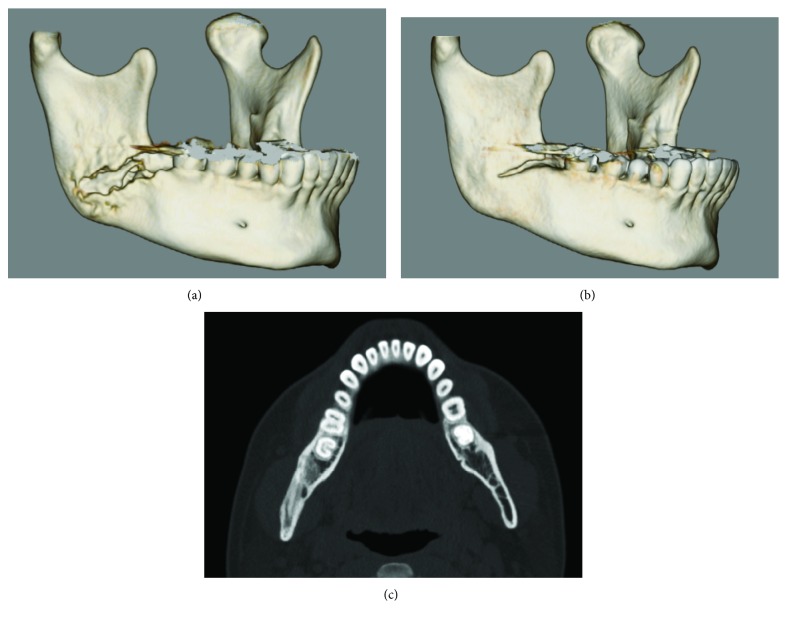
Three-dimensional computed tomographic (3D CT) scan obtained during the initial visit (a). 3D CT (b) and coronal CT (c) scans obtained 6 months after sequestrectomy and curettage. Although the CT obtained at the initial visit shows destruction of buccal cortical bone from the socket to the right mandibular angle and formation of bone sequestrum (a), CT obtained at 6 months shows bone healing at the lesion site, except for the remaining slight slit of the buccal cortical bone (b, c).

After a clinical diagnosis of osteomyelitis of the mandible caused by late fracture following third molar extraction, we performed sequestrectomy and curettage via a mandibular vestibular approach under general anesthesia in April 2018, followed by administration of ampicillin/sulbactam for 5 days. After flap elevation, the granulation tissue was eliminated using a sharp curette, and the edges of the remaining bone were rounded off. The sequestrum detected by CT obtained during the initial visit completely disappeared and replaced by immature granulation tissues. The size of the defect of the buccal cortical bone was 25 mm (height) × 8 mm (width) × 6 mm (depth), which reached from the extraction socket to the right mandibular angle (Figure 6). Given that the lingual cortical bone was intact, there was no mobility and an inferior alveolar nerve was not exposed to the surgical field. Pathological findings of the surgical specimen revealed that the granulation tissue had drusen of colonies of *Actinomyces* sp. and gram-positive bacillus (Figures 7(a)–7(c)). The trismus and paresthesia of his lower lip and mental region in the right side disappeared completely, and no relapse occurred during the 6-month follow-up (Figures 8(a)–8(c)).

## 3. Discussion

This is a rare and valuable report describing a case of osteomyelitis of the mandible caused by late fracture following third molar extraction. To prevent postoperative mandibular fracture, patients undergoing third molar extraction should maintain a liquid and soft diet and should expect to return to their regular physical activities at four weeks after the procedure [9–14]. Generally, postoperative fractures occurred more common in Class II/III and Type B/C impaction than in Class I and Type A impactions, and patients older than 30-40 years with a full dentition were considered to be a risk group [2, 9, 13, 15, 16]. Moreover, most fractures associated with the extraction of teeth arose postoperatively and usually occurred during second to fourth week [9–12, 14, 17]. Interestingly, a number of fractures occurred during chewing of bagel, nuts, and steak [11]. The masticatory force required to break down food before deglutition can place considerable stress on bone weakened by surgery, which is not yet fully restored [6, 11]. However, preangular courses of fracture have been described as typical for pathological mandibular fractures following third molar removal [2]; Wagner et al. [13] reported two angular courses of fracture.

Early and adequate clinical and radiologic evaluations are required to establish a correct diagnosis of postoperative mandibular fracture. Typically, a cracking sound from the mandible while eating frequently indicated that a fracture had occurred [2, 12, 16]. Then, following the cracking sound, a swelling in the respective mandibular angle was frequently detectable [13]. Given that the course of the patients until this episode had been uneventful, the mandible was considered to have been fractured at the time this cracking sound was heard. On the other hand, initial radiographs may not be able to reveal a fracture, because the majority of patients do not experience occlusal displacement [2, 10]. In cases where the radiographic finding is negative but a nondisplaced mandibular fracture is suspected, CT should be performed [10]. In such a situation, a soft diet should be prescribed, and repeated radiological examinations are indicated several days later for definitive diagnosis [2].

The treatment options for this type of fracture are diverse and include conservative treatment, a postoperative diet of soft food for several months, maxillomandibular fixation with elastics, and open reduction with internal fixation [11, 13]. Particularly, Pires et al. [10] reported in a systemic review that patients with nondisplaced fractures who were prescribed with a soft diet had a successful treatment and achieved bone repair, as observed radiographically. In the present case, the postoperative course was uneventful, but at 23 days after the extraction, when the patient was chewing an innards stew, he heard a cracking sound from the right mandible and began to feel mild pain and swelling in the right masseteric region. Furthermore, he had paresthesia of his lower lip and mental region in the right side in addition to worsening of the pain and swelling at 19 days after the cracking sound. Even in imaging studies, a panoramic radiograph taken immediately after the cracking sound showed no abnormalities, and CT obtained during the initial visit showed buccal cortical bone destruction from the socket to the right mandibular angle and bone sequestrum formation.

From these findings and course, the patient was diagnosed as having osteomyelitis of the mandible caused by late fracture following third molar extraction. First, in brief, fissure fracture of the buccal cortical bone at the right mandibular angle without dislocation occurred when he heard the cracking sound. These results are consistent with previous reports on the common period, typical trigger, and findings of imaging studies. Then, the infection progressed along the fracture line and extended to the mandibular angle, and osteomyelitis occurred. Finally, the paresthesia of his lower lip and mental region in the right side associated with the osteomyelitis occurred 19 days after the cracking sound. In a past report, Iizuka et al. [2] reported a case of mandibular fracture following third molar extraction with an infection at the fracture site during intermaxillary fixation and required removal of granulation tissue. Therefore, it is necessary to recognize the possibility that late fracture following third molar extraction can cause osteomyelitis. In many cases, late fractures are treated with conservative approaches, such as placing the patients on a soft diet. However, regular and strict monitoring should still be performed during the period of conservative therapy to prevent the development of osteomyelitis following a late fracture, as seen in this case.

Second, once osteomyelitis of the mandible caused by late fracture occurred, early and appropriate treatment is necessary because the osteomyelitis develops rapidly, and there is a possibility that it will result in pathological fracture. The bacteria associated with osteomyelitis includes *Staphylococcus* sp., *Peptostreptococcus* sp., *Actinomyces* sp., and *Prevotella* sp. [18–20]. Once seeded, the infections are thought to spread via the medullary marrow space and compromise the blood supply. The affected bone is destroyed at a rapid rate in most suppurative cases with formation of sequestra and involucrum [19]. Necrotic tissue promotes the proliferation of bacteria, which, without an appropriate intervention, will result in incomplete healing and progression of the osteomyelitis [21]. In chronic forms of osteomyelitis, the inflammatory infiltrate is composed of plasma cells, lymphocytes, and macrophages, and reactive bone formation is evident with irregular reversal lines [19]. Given that antibiotics cannot penetrate in the affected bone, an early diagnosis of osteomyelitis and a surgical intervention are essential to avoid serious complications [21, 22]. In the present case, new bone formation by periosteal reaction and mixed infection with *Actinomyces* sp. and gram-positive bacilli occurred. However, CT obtained at 31 days after the cracking sound showed buccal cortical bone destruction from the socket to the right mandibular angle and bone sequestrum formation; the sequestrum was completely replaced by granulation tissues at the time of surgery, which was 41 days after the CT was performed. Given the findings showing the rapid progression of osteomyelitis, if early and appropriate treatment was not performed, there was a possibility that the osteomyelitis may have progressed further and pathological fracture resulting from lingual cortical bone fracture may have occurred.

As a limitation of this case report, we cannot completely rule out the possibility that there was already osteomyelitis of the mandible at the time of the cracking sound and a simple pathological fracture occurred. Thus, further studies on cases of late fracture following third molar extraction are required to confirm whether late fractures can cause osteomyelitis.

## 4. Conclusion

The course of this patient has two important implications. First, it is necessary to recognize the possibility that late fracture following third molar extraction can cause osteomyelitis. In many cases, late fractures are treated with conservative approaches, such as placing the patients on a soft diet. However, regular and strict monitoring should still be performed during the period of conservative therapy to prevent the development of osteomyelitis following a late fracture, as seen in this case. Second, once osteomyelitis of the mandible caused by late fracture occurred, early and appropriate treatment is necessary because osteomyelitis may progress rapidly and in some cases may result in pathological fracture.
